# A multidimensional muscle-fat-metabolism model for severity stratification in COPD: a multi-center cross-sectional study

**DOI:** 10.3389/fnut.2026.1835742

**Published:** 2026-08-24

**Authors:** Qianlian Xu, Anjie Yao, Hengxing Sun, Yueyuan Shi, Yunlu Gu, Zhong Feng, Ke Hao, Limin Cao, Shuanshuan Xie, Yunfeng Zhang

**Affiliations:** 1 Department of Respiratory Medicine, Shanghai Tenth People’s Hospital, School of Medicine, Tongji University, Shanghai, China; 2 Department of Clinical Laboratory, Shanghai Tenth People’s Hospital, School of Medicine, Tongji University, Shanghai, China; 3 Department of Respiratory and Critical Care Medicine, Shanghai Tongji Hospital, School of Medicine, Tongji University, Shanghai, China; 4 Department of Thoracic Surgery, Shanghai Pulmonary Hospital, School of Medicine, Tongji University, Shanghai, China; 5 State Key Laboratory of Pollution Control and Resource Reuse, Tongji University, Shanghai, China; 6 Department of Respiratory and Critical Care Medicine, The Second People’s Hospital of Lianyungang & The Oncology Hospital of Lianyungang, Lianyungang, China; 7 Department of Respiratory and Critical Care Medicine, Liqun Hospital, Shanghai, China

**Keywords:** body composition, chronic obstructive pulmonary disease (COPD), muscle-fat-metabolism framework, sarcopenia, visceral fat (VFA)

## Abstract

**Background:**

Chronic obstructive pulmonary disease (COPD) is a systemic disorder characterized by significant body composition alterations, including muscle loss (sarcopenia) and abnormal fat distribution. These changes contribute to a high metabolic burden, with increased resting energy expenditure and systemic inflammation accelerating disease progression. Traditional assessments like body mass index (BMI) fail to capture these complex interactions. This study aimed to evaluate a multidimensional “muscle-fat-metabolism” framework for stratifying COPD risk and severity.

**Methods:**

In this multi-center, cross-sectional study, 377 participants (213 stable COPD patients, 164 healthy controls) underwent bioelectrical impedance analysis (BIA) to assess body composition parameters, including fat-free mass index (FFMI), appendicular skeletal muscle mass index (ASMI), visceral fat area (VFA), and basal metabolic rate (BMR) and other indicators. Spirometry was used to confirm COPD diagnosis and grade severity according to GOLD criteria. Statistical analyses included between-group comparisons, correlation analyses, and multivariate logistic regression to develop predictive models.

**Results:**

COPD patients exhibited significantly lower skeletal muscle mass (FFMI, ASMI) and higher systemic fat levels (FM, BFP) compared with controls. Logistic regression analysis suggested that FFMI and ASMI may be protective against COPD risk and severity, whereas FM and BFP may be risk factors for it. Appendicular and truncal fat decreased progressively with advancing GOLD stage. The integrated muscle-fat-metabolism model achieved excellent predictive performance for severe COPD. Body composition and metabolic markers substantially improved risk stratification in patients with COPD.

**Conclusion:**

An integrative assessment of muscle, fat, metabolic, inflammatory, and functional indicators provided a more effective tool for stratifying COPD risk and severity than conventional metrics. This framework facilitates early identification of high-risk phenotypes like sarcopenic obesity and supports the development of personalized management strategies for COPD patients.

## 1 Introduction

Chronic obstructive pulmonary disease (COPD) is a prevalent chronic respiratory disorder characterized by persistent respiratory symptoms and irreversible airflow limitation (1). It affects over 200 million people globally and is a leading cause of mortality, underscoring the urgency of elucidating its pathophysiology (2, 3). The disease is primarily driven by chronic airway and alveolar inflammation, often due to prolonged exposure to noxious particles such as cigarette smoke (4).

In addition to respiratory impairment, COPD is now recognized as a systemic disorder involving significant alterations in body composition (5, 6). Body composition assessment, which partitions body mass into fat mass (FM) and fat-free mass (FFM), is crucial for understanding disease progression and nutritional management in chronic diseases (7). In COPD, alterations in body mass index (BMI), FM, FFM, and body fat percentage (BFP) arise from multifactorial interactions between environmental exposures and genetic predispositions, leading to physiological disturbances such as chronic hypoxia, inflammation, and energy imbalance (8). These alterations significantly reduce physical fitness and quality of life, and are established factors in COPD progression and complication risk (1).

Sarcopenia, characterized by progressive loss of muscle mass and strength, is highly prevalent in COPD patients and manifests as low fat-free mass index (FFMI) and appendicular skeletal muscle mass index (ASMI). It is significantly associated with impaired pulmonary function, reduced exercise capacity, and increased mortality risk (9, 10). In parallel, visceral adiposity, commonly assessed by elevated waist circumference (WC) and visceral fat area (VFA) (11), exacerbates systemic inflammation and metabolic disturbances (12). Crucially, these pathophysiological changes often coexist and may interact synergistically, accelerating COPD progression.

Previous studies have established the importance of sarcopenia and nutritional status in COPD prognosis, but many studies have focused on isolated metrics or integrated fat metrics (e.g., muscle mass or fat mass alone) or relied on conventional indices such as BMI (1). Although these approaches are clinically useful, they may not fully capture the coexistence of muscle depletion, fat redistribution, metabolic disturbance, inflammation, and functional decline in COPD. Reliance on single metrics may also miss mixed phenotypes, such as sarcopenic obesity, that are clinically relevant but difficult to characterize using BMI alone (13). Therefore, the combined and potentially interactive effects of muscle, fat, metabolic, inflammatory, and functional indicators on COPD severity remain insufficiently defined.

To address these gaps, this study employs a multi-center, cross-sectional design within a multidimensional *muscle-fat-metabolism* framework to simultaneously evaluate body composition, spirometry, inflammatory status, and functional indicators. We hypothesize that integrated parameters exhibit synergistic effects on disease severity. The dual objectives are to identify key interactions among these parameters and to develop a clinical model for stratifying high-risk patients. By extending beyond prior associative studies, our approach aims to elucidate complex relationships and construct a practical tool for personalized COPD management. The ultimate goal is to optimize diagnostic and therapeutic strategies, thereby improving patient outcomes.

## 2 Materials and methods

### 2.1 Study design and participants

**FIGURE 1:**
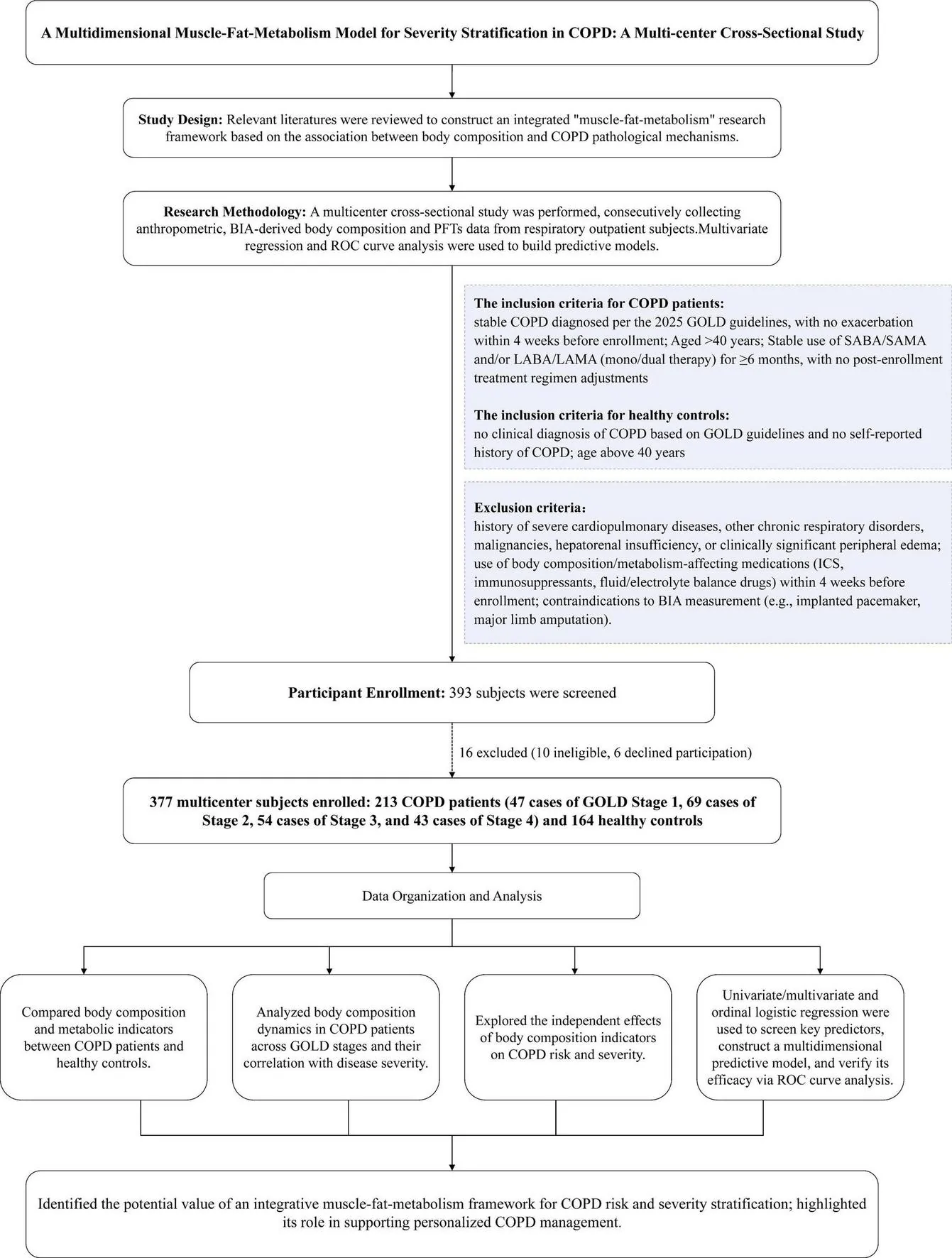
Diagram of research process and patients grouping. COPD, chronic obstructive pulmonary disease; GOLD, Global Initiative for Chronic Obstructive Lung Disease; SABA, Short-Acting β_2_-Agonist; SAMA, Short-Acting Muscarinic Antagonist; LABA, Long-Acting β_2_-Agonist; LAMA, Long-Acting Muscarinic Antagonist; ICS, inhaled corticosteroids; BIA, bioelectrical impedance analysis.

This multi-center, cross-sectional, observational study was conducted from January 2021 to May 2024 at three participating hospitals in China: Shanghai Tenth People’s Hospital, Putuo Liqun Hospital, and Lianyungang Second People’s Hospital. We consecutively recruited stable COPD patients alongside age-matched healthy controls from respiratory outpatient clinics. The detailed enrollment flowchart can be found in Figure 1.

The inclusion criteria for COPD patients were as follows: (1) a diagnosis of stable COPD based on the Global Initiative for Chronic Obstructive Lung Disease (GOLD) guidelines (14), with no exacerbation occurring within the 4 weeks prior to enrollment; (2) age above 40 years; and (3) stable use of Short-Acting β_2_-Agonist (SABA)/Short-Acting Muscarinic Antagonist (SAMA) and/or Long-Acting β_2_-Agonist (LABA)/Long-Acting Muscarinic Antagonist (LAMA) monotherapy or dual therapy for at least 6 months, with no changes in the treatment regimen following enrollment. The exclusion criteria were as follows: (1) a history of severe cardiopulmonary diseases, other chronic respiratory disorders, malignancies, renal dysfunction, or hepatic insufficiency, as well as the presence of clinically significant peripheral edema; (2) administration of medications known to influence body composition or metabolic status, including systemic or inhaled corticosteroids, immunosuppressants, diuretics, or other drugs affecting fluid/electrolyte balance, within the 4 weeks preceding enrollment; and (3) any contraindication to bioelectrical impedance analysis (BIA) measurement, such as the presence of an implantable pacemaker or major limb amputations. For healthy controls, the inclusion criteria were: (1) no clinical diagnosis of COPD based on GOLD guidelines and no self-reported history of COPD; (2) age above 40 years. All exclusion criteria applied to the COPD patients were also applied to the healthy controls.

This study protocol was approved by the Institutional Ethics Committee (Approval No. RT202101) and registered with the Medical Research Registration System (Registration No. MR-31-24-026212, accessible at *medicalresearch.org.cn*). This study was supported by the Shanghai Municipal Health Commission Clinical Research Grant (Grant No. 202140197). Prior to enrollment, all participants provided written informed consent. The study procedures were conducted in accordance with the ethical principles outlined in the Declaration of Helsinki. All participants underwent structured interviews, anthropometric measurements, C-reactive protein (CRP) examination, BIA, pulmonary function tests (PFTs), six-minute walk distance (6MWD) testing, International Physical Activity Questionnaire (IPAQ) assessment, and handgrip strength (HGS) measurement.

A prospective power analysis was performed using G*Power (version 3.1) to determine the minimum sample size required. With an effect size of 0.40, a significance level (α) of 0.05, and a statistical power (1-β) of 0.90, the analysis indicated a requirement of 133 participants per group. The final study cohort comprised 377 individuals (213 patients with COPD and 164 healthy controls), which exceeded the minimum sample size calculated.

### 2.2 Anthropometric and body composition measurements

Anthropometric assessments were performed according to the World Health Organization (WHO) standardized protocols. Height was measured to the nearest 0.01 m, weight to the nearest 0.1 kg, and WC to the nearest 0.1cm. The waist-to-height ratio (WtHR) was calculated as WC divided by height, and the waist-to-hip ratio (WHR) as WC divided by hip circumference. BMI was derived as weight in kilograms divided by the square of height in meters (kg/m^2^). Participants were categorized based on BMI according to established criteria (15): underweight (BMI <18.5 kg/m^2^), normal weight (BMI 18.5-24.99 kg/m^2^), pre-obese (BMI 25-29.99 kg/m^2^), obese class I (BMI 30-34.99 kg/m^2^), obese class II and III (BMI ≥35kg/m^2^).

Body composition was assessed using a BIA device (model IOI353, Beijing Xinruikang, China). The device was calibrated daily using manufacturer-provided phantoms, and inter-operator consistency was ensured through standardized training, achieving an interclass correlation coefficient (ICC) > 0.95 in pilot tests. Following the manufacturer’s protocol, participants were instructed to fast for at least 8 h, refrain from vigorous exercise and caffeinated beverages for 12 h prior to testing, and void their bladder immediately before measurements.

The BIA-derived parameters included BMI, FM, FFM, soft lean mass (SLM), skeletal muscle mass (SMM), appendicular skeletal muscle mass (ASM), trunk soft lean mass (TSLM), BFP, VFA, WC, appendicular fat mass (AFM), trunk fat mass (TFM), basal metabolic rate (BMR), total body water (TBW), extracellular water (ECW) ratio, body cell mass (BCM), estimated protein and mineral content. FM was calculated as body weight minus FFM. The fat mass index (FMI) and FFMI were derived as FM/height^2^ (kg/m^2^) and FFM/height^2^ (kg/m^2^), respectively. The ASMI was calculated as ASM/height^2^ (kg/m^2^). Low FFMI was defined as < 17 kg/m^2^ for males and < 15 kg/m^2^ for females (16). Low ASMI, as determined by BIA, was defined as < 7.0 kg/m^2^ in males and < 5.7 kg/m^2^ in females. Central obesity was defined as a WC ≥ 90 cm for males and ≥ 85 cm for females, and high VFA was defined as ≥ 100 cm^2^.

### 2.3 Pulmonary function testing

PFTs were conducted to measure forced expiratory volume in 1 s (FEV_1_), forced vital capacity (FVC), and the FEV_1_/FVC ratio, in strict compliance with international standard operating procedures. All spirometric assessments were performed by three certified respiratory technicians using identical spirometer models (MasterScreen PFT, CareFusion). Prior to the study, each technician had completed over 50 supervised tests and demonstrated an intra-operator coefficient of variation below 5% in repeated measurements. The results are expressed as percentages of the predicted normal values, derived from Global Lung Function Initiative (GLI) reference equations. COPD severity was graded based on the GOLD criteria (14) as follows: mild (FEV_1_ ≥ 80% predicted), moderate (FEV_1_ 50–79% predicted), severe (FEV_1_ 30–49% predicted), and very severe (FEV_1_ < 30% predicted), with all grades requiring a post-bronchodilator FEV_1_/FVC ratio of < 0.7.

### 2.4 Six-minute walk distance testing

Functional exercise capacity was evaluated using the 6MWD test performed in accordance with American Thoracic Society (ATS) guidelines. Participants were instructed to walk at their maximal sustainable pace for 6 min. The test was terminated immediately if the participant experienced severe discomfort, including chest pain or intolerable dyspnea.

### 2.5 Physical activity assessment

Physical activity was assessed at baseline using the self-reported short-form IPAQ. Total physical activity was calculated and expressed as metabolic equivalent minutes per week (MET-min/week).

### 2.6 Hand grip strength measurement

HGS was measured in the dominant hand using a digital grip dynamometer (YILIAN, BCA-2A, Shanghai, China) following the standard protocol of the General Administration of Sport of China. Three repeated measurements were obtained with a 1-min rest interval between trials, and the maximum value was used for statistical analysis.

### 2.7 Statistical analysis

Missing data were handled using multiple imputation by chained equations, with five imputations performed. All primary variables had a missingness rate of < 5%. Sensitivity analyses comparing results from complete case analysis and the imputed datasets showed consistent findings (*P* > 0.05 for all comparisons). The normality of continuous variables was assessed using the Shapiro-Wilk test. Data are presented as median (interquartile range, IQR) for non-normally distributed variables and mean ± standard deviation (SD) for normally distributed variables. Categorical variables are summarized as frequencies and percentages.

Between-group comparisons were conducted using the Mann-Whitney U test for non-normally distributed continuous variables, independent student’s *t*-test for normally distributed continuous variables, and the Chi-square (χ^2^) test or Fisher’s exact test for categorical variables, as appropriate. For comparisons across multiple GOLD stages, the Kruskal-Wallis test or one-way analysis of variance (ANOVA) was employed for continuous variables, followed by Bonferroni or Dunn *post-hoc* tests. The Cochran-Armitage trend test was used for categorical variables across ordered stages.

Correlations between body composition parameters and COPD risk or disease severity were assessed using Spearman’s rank correlation coefficient (r). Correlation strengths were classified as follows: |*r*| ≥ 0.3 for strong, 0.2–0.3 for moderate, and < 0.2 for weak correlations.

Regression analyses included: (1) univariate and multivariate logistic regression, adjusted for age, sex, smoking, CRP and 6MWD to identify factors associated with COPD occurrence, with results reported as odds ratios (OR) and their 95% confidence intervals (CI); and (2) ordinal logistic regression to analyze associations with GOLD staging, incorporating variables from *muscle-fat-metabolism* models; and (3) exploratory sex-stratified logistic and ordinal logistic analyses using sex-specific sarcopenia thresholds. The predictive performance of the models was evaluated using receiver operating characteristic (ROC) curve analysis. The area under the curve (AUC), sensitivity, specificity, and Youden’s index were calculated for individual models and their composite models. Variable selection was guided by the Akaike Information Criterion (AIC) and a stepwise regression approach. Multicollinearity was assessed using variance inflation factors (VIF), with a VIF value of < 10 considered acceptable.

All statistical analyses were performed using SPSS (version 26.0; IBM Corp.) and R (version 4.2.1; R Foundation). A two-tailed *P*-value of < 0.05 was considered statistically significant, with appropriate corrections (Bonferroni or Dunn) applied for multiple comparisons.

## 3 Results

### 3.1 Baseline characteristics and differences in body composition

A total of 393 subjects were screened, with 377 finally enrolled (213 patients with COPD and 164 healthy controls), and 16 excluded (10 failing to meet the inclusion criteria and 6 refusing participation). The subject flow chart is presented in Figure 1. According to the GOLD criteria, the COPD patients were stratified into Stage 1 (*n* = 47), Stage 2 (*n* = 69), Stage 3 (*n* = 54) and Stage 4 (*n* = 43).

Table 1 summarizes the baseline characteristics, body composition indicators, inflammatory marker, and functional variables of COPD patients and healthy controls. No statistically significant differences were observed in the proportion of males, age or smoking rate between the healthy control group and the COPD group (all *P* > 0.05). There was no significant difference in age across different GOLD stages (*P* > 0.05), whereas the proportion of males and smoking rate differed significantly (both *P* < 0.001), with a gradual increase alongside the elevation of GOLD stage. CRP was significantly higher in the COPD group than in the healthy control group, whereas 6MWD was significantly lower in the COPD group (all *P* < 0.001), with similar trends observed across GOLD stages (all *P* < 0.05).

**TABLE 1:** Inter-group and intra-group differences in body composition: COPD patients vs. healthy controls and across GOLD stages.

Variables	Healthy control (*n* = 164)	COPD (*n* = 213)	Statistical values of the two groups, *P*-value	GOLD 1 (*n* = 47)	GOLD 2 (*n* = 69)	GOLD 3 (*n* = 54)	GOLD 4 (*n* = 43)	Statistical values between multiple groups, *P*-value
Male, *n* (%)	123 (75.00)	173 (81.22)	χ^2^ = 2.13, *P* = 0.145	28 (59.57)	54 (78.26)	49^a^ (90.74)	42^ab^ (97.67)	χ^2^ = 25.68, *P* < 0.001
Age (year)	68.00 (64.00, 75.00)	70.00 (66.00, 73.00)	*Z* = −0.30, *P* = 0.762	71.00 (67.50, 74.00)	69.00 (65.00, 72.00)	69.50 (66.25, 74.00)	68.00 (66.00, 70.00)	χ^2^ = 6.02#, *P* = 0.111
Smoking, *n* (%)	51 (31.10)	71 (33.30)	χ^2^ = 0.21, *P* = 0.646	30 (63.83)	55 (79.71)	45^a^ (83.33)	43^a,b,c^ (100.00)	χ^2^ = 19.52, *P* < 0.001
CRP (mg/L)	1.92 (1.42, 2.79)	2.95 (1.85, 4.65)	*Z* = −5.77, *P* < 0.001	2.36 (1.44, 3.64)	2.96 (1.87, 4.83)	2.70 (1.69, 3.92)	3.90^a,b,c^ (2.51, 5.46)	χ^2^ = 10.92#, *P* = 0.012
6MWD (m)	776.00 (509.00, 1188.00)	560.00 (330.00, 767.00)	*Z* = −5.86, *P* < 0.001	733.00 (592.00, 1100.00)	640.00^a^ (427.00, 812.00)	430.00^a,b^ (324.00, 626.00)	274.00^a,b,c^ (175.00, 462.50)	χ^2^ = 49.46#, *P* < 0.001
BMI (kg/m^2^)	24.13 (21.41, 26.16)	23.66 (20.59, 25.81)	*Z* = −1.31, *P* = 0.191	24.70 (21.60, 26.60)	24.10 (21.70, 25.90)	22.80 (20.30, 25.35)	23.00^a^ (19.05, 24.50)	χ^2^ = 11.94#, *P* = 0.008
FFM (kg)	49.55 (42.10, 54.10)	44.60 (40.90, 48.90)	*Z* = −4.32, *P* < 0.001	45.40 (38.45, 49.95)	45.30 (42.10, 50.50)	45.10 (41.25, 49.03)	43.60^b^ (40.80, 47.40)	χ^2^ = 4.30#, *P* = 0.231
FFMI (kg/m^2^)	17.70 (16.12, 19.02)	16.06 (14.78, 17.46)	*Z* = −6.51, *P* < 0.001	16.48 (15.34, 17.71)	16.42 (15.09, 17.65)	15.84 (14.80, 17.25)	15.28^ab^ (14.25, 16.12)	χ^2^ = 13.85#, *P* = 0.003
Low FFMI, *n* (%)	37 (22.56)	129 (60.56)	χ^2^ = 54.30, *P* < 0.001	20 (42.55)	35 (50.72)	36^b^ (66.67)	38^abc^ (88.37)	χ^2^ = 23.94, *P* < 0.001
SLM (kg)	45.75 (38.85, 50.30)	41.30 (37.70, 45.40)	*Z* = −4.38, *P* < 0.001	41.80 (35.15, 46.20)	41.70 (38.70, 46.60)	41.50 (38.12, 45.20)	40.60 (37.85, 43.95)	χ^2^ = 3.24#, *P* = 0.356
SMM (kg)	27.45 (23.28, 30.20)	23.90 (21.70, 26.50)	*Z* = −5.83, *P* < 0.001	24.60 (20.55, 27.25)	24.50 (22.70, 27.50)	24.00 (22.00, 26.20)	23.10^ab^ (21.40, 25.05)	χ^2^ = 8.15#, *P* = 0.043
ASM (kg)	20.06 (16.62, 22.42)	17.85 (15.84, 20.04)	*Z* = −4.16, *P* < 0.001	18.55 (15.19, 20.61)	18.31 (16.96, 20.72)	18.07 (15.95, 19.71)	16.51^abc^ (15.24, 18.39)	χ^2^ = 11.50#, *P* = 0.009
ASMI (kg/m^2^)	7.16 (6.33, 7.82)	6.45 (5.78, 7.04)	*Z* = −6.12, *P* < 0.001	6.70 (5.94, 7.25)	6.74 (6.06, 7.16)	6.45 (5.65, 6.92)	5.79^ab^ (5.44, 6.21)	χ^2^ = 26.08#, *P* < 0.001
Low ASMI, *n* (%)	48 (29.27)	142 (66.67)	χ^2^ = 51.84, *P* < 0.001	24 (51.06)	37 (53.62)	42^ab^ (77.78)	39^ab^ (90.70)	χ^2^ = 24.61, *P* < 0.001
TSLM (kg)	21.87 (19.22, 23.98)	21.49 (19.93, 23.30)	*Z* = −0.79, *P* = 0.429	21.29 (18.11, 23.26)	21.48 (19.94, 23.68)	21.68 (19.82, 23.35)	21.49 (20.55, 23.08)	χ^2^ = 1.11#, *P* = 0.775
FM (kg)	16.80 (12.52, 20.95)	20.00 (15.60, 23.50)	*Z* = −4.06, *P* < 0.001	21.60 (16.55, 24.75)	20.10 (16.70, 23.40)	17.45^a^ (14.05, 23.38)	20.00 (14.95, 23.20)	χ^2^ = 5.87#, *P* = 0.118
FMI (kg/m^2^)	6.36 (4.31, 7.58)	7.42 (5.55, 8.65)	*Z* = −3.58, *P* < 0.001	7.69 (6.42, 9.88)	7.59 (5.85, 8.65)	6.82^a^ (4.89, 8.34)	6.94 (5.22, 8.19)	χ^2^ = 10.24#, *P* = 0.017
BFP (%)	26.12 (19.92, 30.92)	30.71 (25.55, 34.25)	*Z* = −5.53, *P* < 0.001	32.05 (29.55, 38.14)	29.97 (25.93, 33.47)	28.12^a^ (22.95, 32.60)	32.14 (25.80, 34.00)	χ^2^ = 9.93#, *P* = 0.019
VFA (cm^2^)	117.00 (89.75, 154.00)	122.00 (95.00, 152.00)	*Z* = −1.09, *P* = 0.274	125.34 ± 53.97	122.22 ± 35.55	127.04 ± 49.17	126.93 ± 39.96	*F* = 0.15, *P* = 0.926
High VFA, *n* (%)	104 (63.41)	153 (71.83)	χ^2^ = 3.02, *P* = 0.082	30 (63.83)	52 (75.36)	39 (72.22)	32 (74.42)	χ^2^ = 2.06, *P* = 0.560
WC (cm)	85.90 (78.12, 91.38)	86.00 (80.10, 92.70)	*Z* = −1.17, *P* = 0.243	85.00 (80.25, 92.90)	85.50 (80.20, 92.20)	86.90 (79.75, 91.42)	88.50 (80.50, 92.80)	χ^2^ = 0.29#, *P* = 0.962
Central obesity group, *n* (%)	60 (36.59)	78 (36.62)	χ^2^ = 0.00, *P* = 0.995	19 (40.43)	22 (31.88)	19 (35.19)	18 (41.86)	χ^2^ = 1.52, *P* = 0.678
AFM (kg)	7.48 (5.65, 8.91)	7.46 (5.68, 8.93)	*Z* = −0.23, *P* = 0.815	8.26 (6.21, 9.73)	7.73 (6.22, 9.07)	6.65^a^ (4.69, 8.75)	6.87^ab^ (4.80, 8.09)	χ^2^ = 16.09# *P* = 0.001
TFM (kg)	8.19 (5.89, 10.50)	8.06 (5.48, 10.03)	*Z* = −1.03, *P* = 0.301	9.10 (6.94, 10.54)	8.33 (6.64, 10.15)	6.22^ab^ (4.17, 9.46)	6.93^ab^ (4.51, 8.69)	χ^2^ = 17.90#, *P* < 0.001
BMR (kcal)	1140.00 (1279.00, 1538.00)	1411.00 (1329.00, 1508.00)	*Z* = −0.51, *P* = 0.608	1381.83 ± 166.84	1420.68 ± 154.39	1423.85 ± 164.76	1417.53 ± 123.83	*F* = 0.79, *P* = 0.499
TBW (L)	35.65 (30.78, 39.28)	34.70 (31.90, 38.10)	*Z* = −0.56, *P* = 0.574	34.70 (29.65, 38.00)	34.40 (32.30, 38.40)	35.05 (32.32, 37.98)	34.70 (33.00, 37.75)	χ^2^ = 0.93#, *P* = 0.818
ECW ratio (%)	0.401 (0.396, 0.407)	0.402 (0.396, 0.409)	*Z* = −1.76, *P* = 0.079	0.402 (0.398, 0.411)	0.400 (0.396, 0.406)	0.402 (0.397, 0.405)	0.400 (0.397, 0.404)	χ^2^ = 3.63#, *P* = 0.304
BCM (kg)	33.20 (28.23, 36.20)	32.30 (29.60, 35.40)	*Z* = −3.27, *P* = 0.001	32.10 (26.90, 35.25)	31.90 (29.60, 35.60)	32.70 (30.15, 35.58)	32.70 (30.55, 35.20)	χ^2^ = 1.54#, *P* = 0.672
Protein (kg)	9.00 (7.60, 10.70)	9.10 (8.30, 10.00)	*Z* = −0.41, *P* = 0.682	9.32 ± 1.67	9.33 ± 1.50	9.01 ± 1.58	8.99 ± 1.15	*F* = 0.85, *P* = 0.469
Minerals (kg)	3.50 (3.20, 3.80)	3.50 (3.30, 3.70)	*Z* = −0.16, 6 = 0.869	3.50 (3.10, 3.70)	3.50 (3.30, 3.90)	3.50 (3.30, 3.80)	3.50 (3.40, 3.70)	χ^2^ = 4.41#, *P* = 0.220
HGS (kg)	36.5 ± 6.17	35.8 ± 6.23	*Z* = −0.89, *P* = 0.278	36.2 ± 6.14	35.6 ± 6.38	35.4 ± 6.20	35.1 ± 6.31	*F* = 0.25, *P* = 0.862
IPAQ (MET-min/week)	2910 ± 3043	2884 ± 2925	*Z* = −0.18, *P* = 0.973	2901 ± 2856	2892 ± 2061	2873 ± 2314	2868 ± 2157	*F* = 0.06, *P* = 0.974

*P*-values between two groups were derived from the Mann-Whitney test (for non-normally distributed variables) and chi-square test (for categorical variables). F: ANOVA (normally distributed variables with homogeneous variance); #: Kruskal-Wallis test (for non-normally distributed variables); χ^2^: chi-square test (for categorical variables); *post hoc* pairwise comparisons used Bonferroni correction or Dunn test. Data are presented as mean ± SD, median (IQR 25, 75%), or percentage (%).

a: compared with GOLD grade 1, *P* < 0.05;

b: compared with GOLD grade 2, *P* < 0.05;

c: compared with GOLD grade 3, *P* < 0.05;

d: compared with GOLD grade 4, *P* < 0.05.

CRP, C-Reactive Protein; 6MWD, 6-Min Walk Distance; BMI, body mass index; FFM, fat free mass; FFMI, fat free mass index; SLM, soft lean mass; SMM, skeletal muscle mass; ASM, appendicular skeletal muscle mass; ASMI, appendicular skeletal muscle mass index; TSLM, trunk soft lean mass; FM, fat mass; FMI, fat mass index; BFP, body fat percentage; VFA, visceral fat area; WC, waist circumference; AFM, appendicular fat mass; TFM, trunk fat mass; BMR, basal metabolic rate; TBW, total body water; ECW, extracellular water; BCM, body cell mass; HGS, hand grip strength; IPAQ, International Physical Activity Questionnaire.

For body mass and muscle-related indicators, BMI varied significantly across GOLD stages (*P* = 0.008), with Stage 4 significantly lower than Stage 1 [23.00 (19.05, 24.50) kg/m^2^ vs. 24.70 (21.60, 26.60) kg/m^2^, *P* < 0.05]. FFM and SLM in the COPD group were significantly lower than those in the healthy control group (*P* < 0.001), with no inter-stage differences (*P* > 0.05). FFMI, SMM, ASM and ASMI in the COPD group were all significantly lower than those in the healthy control group (all *P* < 0.001), with significant differences across GOLD stages (*P* < 0.05); additionally, FFMI, SMM, ASM, and ASMI exhibited a decreasing trend with the advancement of GOLD stage.

For fat-related indicators, FM in the COPD group was significantly higher than that in the healthy control group [20.00 (15.60, 23.50) kg vs. 16.80 (12.52, 20.95) kg, *P* < 0.001], with no significant difference across GOLD stages (*P* > 0.05). TFM and AFM showed no inter-group difference (*P* > 0.05) but significant variations across GOLD stages (*P* ≤ 0.001), with lower levels in higher GOLD stages. FMI and BFP differed significantly both between the two groups and across GOLD stages (all *P* < 0.05); both indicators were significantly higher in the COPD group than in the healthy control group (*P* < 0.001), and Stage 3 values were markedly lower than those of Stage 1 (*P* < 0.05).

For metabolic and cellular hydration indicators, BCM in the COPD group was significantly lower than that in the healthy control group [32.30 (29.60, 35.40) kg vs. 33.20 (28.23, 36.20) kg, *P* = 0.001], with no significant difference across GOLD stages (*P* > 0.05). Regarding physical function, the HGS and IPAQ had no significant differences between different groups (all *P* > 0.05).

### 3.2 Correlation between body composition indicators and COPD risk and severity

As shown in Table 2, FM, FMI, CRP and BFP were significantly positively correlated with the risk of COPD (all *P* < 0.001), while 6MWD, FFM, FFMI, SLM, SMM, ASM, ASMI and protein were significantly negatively correlated with COPD risk (all *P* < 0.001). The proportion of males, smoking rate and CRP were significantly positively correlated with COPD severity (both *P* < 0.05), whereas BMI, FFMI, 6MWD, ASM, ASMI, FM, FMI, BFP, AFM, and TFM were significantly negatively correlated with COPD severity (all *P* < 0.05).

**TABLE 2:** Correlation analysis of body composition indicators for COPD risk and severity.

Indicators	COPD patients vs. healthy controls		COPD severity (GOLD 1–4)	
	Spearman correlation coefficient (r)	*P*-value	Spearman correlation coefficient (r)	*P*-value
Male, *n* (%)	0.075	0.146	0.341	< 0.001
Age (year)	0.000	0.994	−0.119	0.083
Smoking, *n* (%)	0.024	0.647	0.293	< 0.001
BMI (kg/m^2^)	−0.060	0.249	−0.237	< 0.001
CRP (mg/L)	0.298	< 0.001	0.190	0.005
6MWD (m)	−0.302	< 0.001	−0.482	< 0.001
Muscle mass indicators				
FFM (kg)	−0.218	< 0.001	−0.069	0.315
FFMI (kg/m^2^)	−0.314	< 0.001	−0.231	< 0.001
Low FFMI, n(%)	0.380	< 0.001	0.325	< 0.001
SLM (kg)	−0.220	< 0.001	−0.055	0.428
SMM (kg)	−0.295	< 0.001	−0.131	0.057
ASM (kg)	−0.186	< 0.001	−0.169	0.014
ASMI (kg/m^2^)	−0.231	< 0.001	−0.308	< 0.001
Low ASMI, n(%)	0.371	< 0.001	0.324	< 0.001
TSLM (kg)	−0.053	0.301	0.048	0.489
Fat related indicators				
FM (kg)	0.205	< 0.001	−0.144	0.036
FMI (kg/m^2^)	0.171	< 0.001	−0.200	0.003
BFP (%)	0.289	< 0.001	−0.138	0.044
VFA (cm^2^)	0.023	0.653	0.067	0.331
High VFA, *n* (%)	0.090	0.082	0.062	0.366
WC (cm)	0.060	0.247	0.019	0.782
Central obesity group, *n*(%)	0.000	0.995	−0.025	0.712
AFM (kg)	−0.005	0.916	−0.273	< 0.001
TFM (kg)	−0.057	0.267	−0.282	< 0.001
Metabolic and cell hydration parameters				
BMR (kcal)	−0.017	0.742	0.069	0.319
TBW (L)	−0.020	0.693	0.045	0.517
ECW ratio (%)	−0.094	0.068	−0.089	0.194
BCM (kg)	−0.011	0.829	0.068	0.326
Protein (kg)	−0.189	< 0.001	−0.121	0.078
Minerals (kg)	0.009	0.855	0.093	0.174

(|r| ≥ 0.3): strong correlation; (0.2 ≤ |r| < 0.3): moderate correlation; (|r| < 0.2): weak correlation. CRP, C-Reactive Protein; 6MWD, 6-Min Walk Distance; BMI, Body mass index; FFM, Fat free mass; FFMI, Fat free mass index; SLM, Soft lean mass; SMM, Skeletal muscle mass; ASM, Appendicular skeletal muscle mass; ASMI, Appendicular skeletal muscle mass index; TSLM, Trunk soft lean mass; FM, Fat mass; FMI, Fat mass index; BFP, Body fat percentage; VFA, Visceral fat area; WC, Waist circumference; AFM, Appendicular fat mass; TFM, Trunk fat mass; BMR, Basal Metabolic Rate; TBW, Total body water; ECW, Extracellular Water; BCM, Body cell mass.

### 3.3 Logistic regression analysis of body composition indicators with COPD risk and severity

To explore the predictive value of body composition indicators for COPD risk and severity, three models were constructed as shown in Table 3: an unadjusted univariate model (Model 1), a multivariate model adjusted for age, sex and smoking (Model 2), and a multivariate model adjusted for age, sex, smoking, CRP and 6MWD (Model 3).

**TABLE 3:** Logistic regression analysis of body composition indicators for COPD risk and severity.

Variables	COPD patients vs. healthy controls^a^						COPD severity (GOLD 1∼ GOLD 4)^b^					
	Model 1 (univariate)		Model 2 (adjusted for age, sex and smoking)		Model 3 (adjusted for age, sex, smoking, CRP and 6MWD)		Model 1 (univariate)		Model 2 (adjusted for age, sex and smoking)		Model 3 (adjusted for age, sex, smoking, CRP and 6MWD)	
	OR (95% CI)	*P*-value	OR (95% CI)	*P*-value	OR (95% CI)	*P*-value	OR (95% CI)	*P*-value	OR (95% CI)	*P*-value	OR (95% CI)	*P*-value
Muscle mass indicators												
FFMI (kg/m^2^)	0.731 (0.657, 0.813)	< 0.001	0.686 (0.610, 0.771)	< 0.001	0.664 (0.586, 0.753)	< 0.001	0.836 (0.738, 0.947)	0.005	0.749 (0.652, 0.861)	< 0.001	0.766 (0.684, 0.856)	< 0.001
Low FFMI, *n*(%)	5.271 (3.335, 8.332)	< 0.001	5.388 (3.387, 8.572)	< 0.001	6.997 (4.131, 11.852)	< 0.001	3.435 (2.051, 5.755)	< 0.001	3.498 (2.051, 5.966)	< 0.001	3.096 (1.783, 5.377)	< 0.001
SLM (kg)	0.942 (0.915, 0.969)	< 0.001	0.893 (0.860, 0.928)	< 0.001	0.881 (0.846, 0.918)	< 0.001	0.991 (0.957, 1.027)	0.626	0.918 (0.877, 0.961)	< 0.001	0.923 (0.889, 0.959)	< 0.001
SMM (kg)	0.873 (0.831, 0.917)	< 0.001	0.788 (0.737, 0.842)	< 0.001	0.773 (0.719, 0.830)	< 0.001	0.956 (0.901, 1.014)	0.132	0.832 (0.770, 0.898)	< 0.001	0.844 (0.791, 0.899)	< 0.001
ASMI (kg/m^2^)	0.654 (0.538, 0.794)	< 0.001	0.585 (0.471, 0.727)	< 0.001	0.567 (0.451, 0.712)	< 0.001	0.668 (0.526, 0.849)	< 0.001	0.512 (0.382, 0.685)	< 0.001	0.539 (0.426, 0.684)	< 0.001
Low ASMI, *n* (%)	4.833 (3.110, 7.511)	< 0.001	5.054 (3.200, 7.980)	< 0.001	5.859 (3.523, 9.743)	< 0.001	3.546 (2.081, 6.043)	< 0.001	2.836 (1.631, 4.929)	< 0.001	2.333 (1.318, 4.129)	0.004
Fat related indicators												
FM (kg)	1.067 (1.033, 1.102)	< 0.001	1.082 (1.045, 1.120)	< 0.001	1.061 (1.023, 1.101)	0.002	0.944 (0.909, 0.981)	0.003	0.969 (0.930, 1.010)	0.136	0.980 (0.939, 1.023)	0.980
BFP(%)	1.084 (1.053, 1.117)	< 0.001	1.149 (1.105, 1.195)	< 0.001	1.128 (1.083, 1.175)	< 0.001	0.952 (0.919, 0.986)	0.006	1.004 (0.961, 1.050)	0.852	1.015 (0.974, 1.057)	0.492
VFA (cm^2^)	1.001 (0.997, 1.005)	0.652	1.000 (0.996, 1.005)	0.910	0.998 (0.994, 1.003)	0.542	1.001 (0.996, 1.007)	0.697	0.998 (0.992, 1.004)	0.452	1.000 (0.994, 1.006)	0.997
High VFA, *n* (%)	1.471 (0.951, 2.275)	0.083	1.384 (0.877, 2.185)	0.163	1.261 (0.769, 2.068)	0.358	1.289 (0.749, 2.220)	0.359	0.821 (0.463, 1.457)	0.501	0.896 (0.497, 1.615)	0.715
WC (cm)	1.014 (0.991, 1.037)	0.246	1.009 (0.985, 1.034)	0.449	0.994 (0.968, 1.021)	0.663	0.997 (0.970, 1.026)	0.862	0.975 (0.946, 1.005)	0.100	0.982 (0.958, 1.007)	0.153
AFM (kg)	0.996 (0.924, 1.073)	0.916	1.017 (0.939, 1.102)	0.675	0.973 (0.892, 1.061)	0.536	0.806 (0.732, 0.887)	< 0.001	0.857 (0.772, 0.951)	0.004	0.871 (0.782, 0.971)	0.012
TFM (kg)	0.969 (0.917, 1.024)	0.267	0.979 (0.923, 1.037)	0.464	0.945 (0.886, 1.008)	0.083	0.853 (0.796, 0.913)	< 0.001	0.879 (0.817, 0.946)	< 0.001	0.902 (0.836, 0.974)	0.008
Metabolic and cell hydration parameters												
BMR (kcal)	1.000 (0.999, 1.001)	0.741	0.999 (0.997, 1.000)	0.134	0.998 (0.996, 0.999)	0.021	1.001 (1.001, 1.001)	< 0.001	0.998 (0.996, 0.999)	0.006	0.998 (0.996, 0.999)	0.011
TBW (L)	0.993 (0.959, 1.028)	0.692	0.968 (0.927, 1.011)	0.139	0.946 (0.903, 0.992)	0.021	1.017 (0.973, 1.062)	0.467	0.939 (0.889, 0.993)	0.026	0.943 (0.901, 0.987)	0.012
Protein (kg)	0.794 (0.701, 0.901)	< 0.001	0.599 (0.500, 0.718)	< 0.001	0.566 (0.466, 0.687)	< 0.001	0.890 (0.757, 1.046)	0.157	0.522 (0.411, 0.661)	< 0.001	0.546 (0.453, 0.658)	< 0.001
Minerals(kg)	1.048 (0.637, 1.725)	0.854	0.776 (0.410, 1.471)	0.437	0.545 (0.268, 1.108)	0.094	1.585 (0.806, 3.116)	0.182	0.469 (0.210, 1.051)	0.066	0.468 (0.269, 0.816)	0.007

*^a^*Univariate or multivariate binary logistic regression analysis of body compositions indicators for COPD occurrence.

*^b^*Univariate or multivariate ordinal logistic regression analysis of body compositions indicators for COPD severity (GOLD 1∼4).

Model 1: univariate analysis; Model 2: adjusted for age, sex and smoking; Model 3: adjusted for age, sex, smoking, CRP and 6MWD. *P*-values indicate statistical significance. The Bootstrap method with 1,000 resamples was used to validate the robustness of the results. CRP, C-Reactive Protein; 6MWD, 6-Min Walk Distance; OR, Odds ratio; CI, Confidence interval; FFMI, Fat free mass index; SMM, Skeletal muscle mass; ASMI, Appendicular skeletal muscle mass index; FM, Fat mass; BFP, Body fat percentage; VFA, Visceral fat area; WC, Waist circumference; AFM, Appendicular fat mass; TFM, Trunk fat mass; BMR, Basal Metabolic Rate; TBW, Total body water.

Among muscle mass indicators, FFMI and ASMI served as protective factors for both COPD risk and severity in all three models (all *P* < 0.05, OR < 1). SLM and SMM were protective factors for COPD risk in all three models (all *P* < 0.001, OR < 1) and also acted as protective factors for COPD severity in Model 2 and Model 3 (all *P* < 0.001, OR < 1).

Among fat-related indicators, FM and BFP were risk factors for COPD in all three models (all *P* < 0.05, OR > 1) and protective factors for COPD severity in only Model 1 (all *P* < 0.05, OR < 1). AFM and TFM were protective factors for COPD severity in all three models (all *P* < 0.05, OR < 1).

Among metabolic and cellular hydration indicators, protein was a protective factor for COPD risk in all three models and only a protective factor for COPD severity in Model 2 and Model 3 (all *P* < 0.001, OR < 1). BMR was a risk factor for COPD severity in Model 1 (*P* < 0.001, OR > 1) and converted to a protective factor in Model 2 and Model 3 (all *P* < 0.05, OR < 1). TBW showed no significant effect in Model 1 (*P* > 0.05) but functioned as a protective factor for COPD severity in Model 2 and Model 3 (all *P* < 0.05, OR < 1).

### 3.4 Predictive efficacy of body composition indicators for COPD risk and severity

**FIGURE 2:**
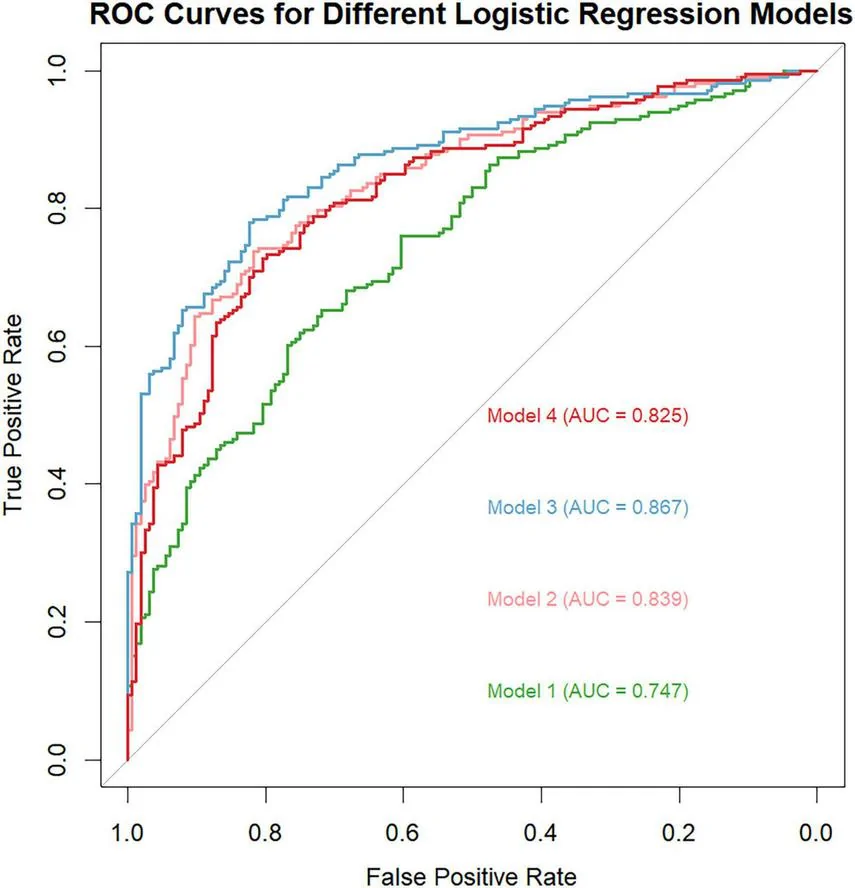
Receiver operating characteristic (ROC) curve analysis for established COPD risk assessment models. Model 1 (Clinical Basic Model) included WC and BMI; Model 2 (Composition Distribution Model) included FFMI and WC; Model 3 (Composition Metabolism Model) included FFMI, WC and BMR; Model 4 (Classification Model) included low ASMI, high VFA and BMR. All the models have adjusted for age, sex, smoking, CRP and 6MWD. AUC, area under the curve; CI, confidence interval; BMI, body mass index; WC, waist circumference; FFMI, fat free mass index; BMR, basal metabolic rate; ASMI, appendicular skeletal muscle mass index; VFA, visceral fat area; CRP, C-reactive protein; 6MWD, 6-minute walk distance.

To optimize the efficacy of COPD risk assessment, four combined logistic regression models were constructed after adjustment for sex, age, smoking, *CRP and* 6MWD (details in Table 4): (1) clinical baseline model (including BMI and WC); (2) composition distribution model (including FFMI and WC); (3) composition metabolism model (including FFMI, WC and BMR); and (4) classification model (including low ASMI, VFA status and BMR). ROC curve analysis (Figure 2 and Supplementary Table 1) revealed that composition metabolism model had the optimal predictive efficacy (AUC = 0.867), followed by the composition distribution model (AUC = 0.839) and the classification model (AUC = 0.825). All three models were significantly superior to the clinical baseline model (AUC = 0.747), suggesting that muscle mass and metabolic characteristics are the core predictive dimensions for COPD risk.

**TABLE 4:** Multivariate logistic regression analysis of body composition indicators for COPD occurrence.

Variables	Multivariate logistic regression analysis (adjusted for age, sex, smoking, CRP and 6MWD)					
	β	S.E	*Z*	*P*	OR (95%CI)	VIF
Model 1: Clinical baseline model						
BMI	−0.195	0.063	−3.073	0.002	0.823 (0.727 ∼ 0.932)	4.506
WC	0.073	0.029	2.503	0.012	1.075 (1.016 ∼ 1.139)	4.919
Model 2: Composition distribution model						
FFMI	−0.666	0.099	−6.732	< 0.001	0.514 (0.423 ∼ 0.624)	1.545
WC	0.092	0.021	4.282	< 0.001	1.096 (1.051 ∼ 1.143)	1.619
Model 3: Composition metabolism model						
FFMI	−1.166	0.147	−7.919	< 0.001	0.312 (0.233 ∼ 0.416)	3.285
WC	0.072	0.022	3.354	< 0.001	1.075 (1.031 ∼ 1.121)	1.688
BMR	0.010	0.002	5.241	< 0.001	1.010 (1.006 ∼ 1.014)	4.972
Model 4: Classification model						
Low ASMI	2.470	0.357	6.920	< 0.001	11.824 (5.874 ∼ 23.803)	1.721
High VFA	0.576	0.286	2.013	0.044	1.780 (1.015 ∼ 3.119)	1.158
BMR	0.003	0.001	2.625	0.009	1.003 (1.001 ∼ 1.005)	2.742

CRP, C-Reactive Protein; 6MWD, 6-Min Walk Distance; OR, Odds ratio; CI, Confidence interval; VIF, Variance inflation factor; BMI, Body mass index; WC, Waist circumference; FFMI, Fat free mass index; BMR, Basal metabolic rate; ASMI, Appendicular skeletal muscle mass index; VFA, Visceral fat area.

**FIGURE 3:**
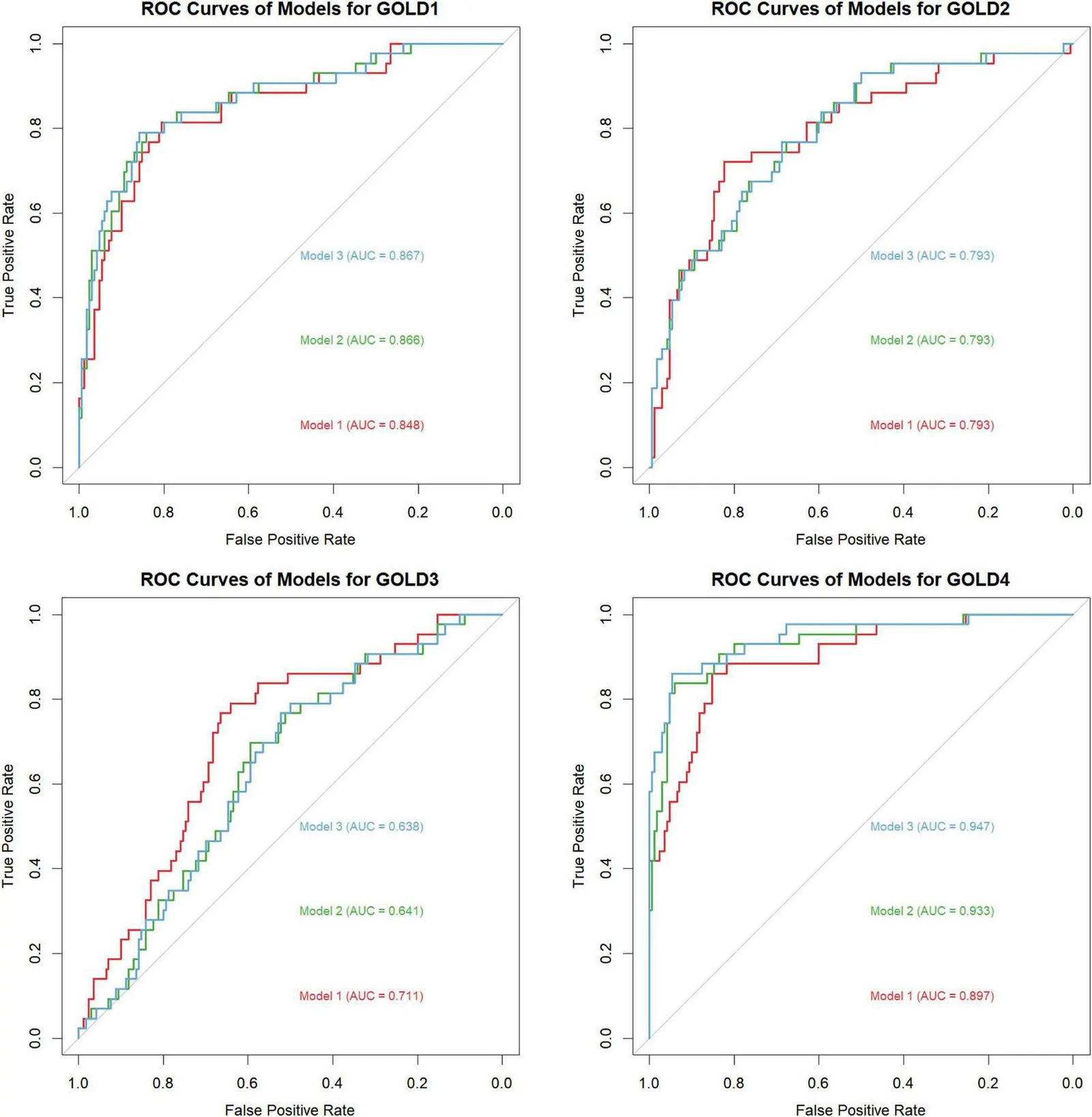
Receiver operating characteristic (ROC) curve analysis for established COPD severity assessment models. Model 1 (Composition Metabolism Model) included FFMI, WC and BMR; Model 2 (Muscle-Fat Model) included ASMI and BFP; Model 3 (Muscle-Fat Metabolism Model) included ASMI, BFP and BMR. All the models have adjusted for age, sex, smoking, CRP and 6MWD. AUC, area under the curve; CI, confidence interval; BMI, body mass index; WC, waist circumference; FFMI, fat free mass index; BMR, basal metabolic rate; ASMI, appendicular skeletal muscle mass index; BFP, body fat percentage; CRP, C-reactive protein; 6MWD, 6-minute walk distance.

**FIGURE 4:**
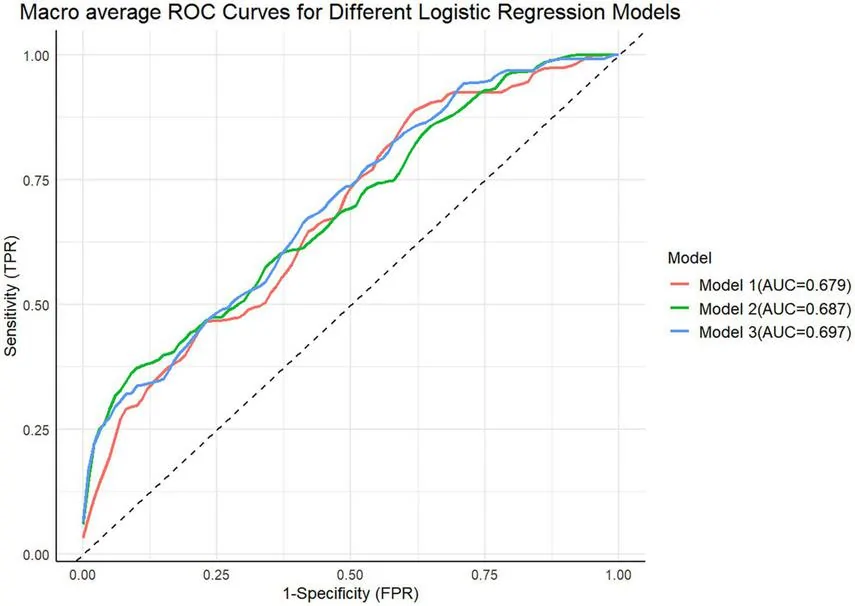
Macro average ROC analysis for established COPD severity assessment models. Model 1 (Composition Metabolism Model) included FFMI, WC and BMR; Model 2 (Muscle-Fat Model) included ASMI and BFP; Model 3 (Muscle-Fat Metabolism Model) included ASMI, BFP and BMR. All the models have adjusted for age, sex, smoking, CRP and 6MWD. AUC, area under the curve; CI, confidence interval; BMI, body mass index; WC, waist circumference; FFMI, fat free mass index; BMR, basal metabolic rate; ASMI, appendicular skeletal muscle mass index; BFP, body fat percentage; CRP, C-reactive protein; 6MWD, 6-minute walk distance.

For the assessment of COPD severity, two multivariate ordinal logistic regression models were established (details in Table 5): muscle-fat model (including ASMI and BFP) and muscle-fat-metabolism model (including ASMI, BFP, and BMR). ROC curve analysis comparing the above models with the composition metabolism model demonstrated that all models had better stratification efficacy for GOLD Stage 4 patients than for those in other stages, with the muscle-fat-metabolism model achieving the highest discriminative efficacy for Stage 4 patients (AUC = 0.947, Figure 3). This indicated that the model integrating body composition and metabolic characteristics had the strongest ability to identify the most severe stage of COPD. In addition, macro-average ROC curve analysis (Figure 4 and Supplementary Table 2) showed that all models had good overall discriminative ability for COPD severity; although the muscle-fat-metabolism model attained a numerically higher macro-average AUC (0.697 vs. 0.687 and 0.679), the differences between models were small and the model was not uniformly superior across the individual GOLD stages, its advantage being most evident for GOLD Stage 4.

**TABLE 5:** Multivariate ordinal logistic regression analysis of body composition indicators for COPD severity (GOLD 1∼4).

Variables	Multivariate ordinal logistic regression analysis (adjusted for age, sex, smoking, CRP and 6MWD)					
	β	S.E	*t*	*P*	OR (95%CI)	VIF
Composition metabolism model						
FFMI	−0.532	0.180	−2.951	0.003	0.588 (0.413 ∼ 0.836)	3.532
WC	0.033	0.023	1.438	0.150	1.033 (0.988 ∼ 1.080)	1.907
BMR	0.003	0.002	1.256	0.209	1.003 (0.998 ∼ 1.008)	4.783
Muscle-fat model						
ASMI	−0.727	0.145	−5.029	< 0.001	0.483 (0.364 ∼ 0.642)	1.116
BFP	0.053	0.025	2.110	0.035	1.054 (1.004 ∼ 1.107)	1.679
Muscle-fat-metabolism model						
ASMI	−1.380	0.426	−3.235	0.001	0.252 (0.109 ∼ 0.580)	1.831
BFP	0.050	0.025	1.986	0.047	1.052 (1.001 ∼ 1.105)	1.706
BMR	0.006	0.003	2.114	0.034	1.006 (1.001 ∼ 1.011)	2.593

CRP, C-Reactive Protein; 6MWD, 6-Min Walk Distance; OR, Odds ratio; CI, Confidence interval; VIF, Variance inflation factor; FFMI, Fat free mass index; WC, Waist circumference; BMR, Basal metabolic rate; ASMI, Appendicular skeletal muscle mass index; BFP, Body fat percentage.

Exploratory sex-stratified subgroup analyses were performed to address the sex-specific diagnostic thresholds for low ASMI and low FFMI. Supplementary Table 3 presents multivariable logistic regression analyses for COPD occurrence stratified by sex, and Supplementary Table 4 presents multivariable ordinal logistic regression analyses for COPD severity (GOLD stages 1–4) stratified by sex. These analyses generally supported the direction of the main findings, particularly the protective role of muscle-related indicators and the male subgroup exhibited a greater number of significant body composition indicators than the female subgroup. However, it’s necessary to collect larger sex subgroup sample sizes to perform more formal interaction testing in the future.

## 4 Discussion

This multi-center study identified a distinct multi-dimensional body composition phenotype in stable COPD, characterized by skeletal muscle depletion, altered fat distribution, systemic inflammation, reduced exercise capacity, and metabolic dysregulation. Crucially, we demonstrate that an integrative “*muscle-fat-metabolism*” framework surpasses conventional metrics like BMI in stratifying COPD risk and severity. These findings underscore the pathophysiological synergy between these body components, as detailed in the following interpretations of muscle, fat and metabolic alterations.

First, our results confirm sarcopenia as a prevalent and progressive systemic manifestation in COPD, evidenced by significantly lower FFMI, SMM, ASM, and ASMI compared to healthy controls and a further decline across advancing GOLD stages (17, 18). This muscle loss is the combined result of systemic inflammation, oxidative stress, hypercatabolism, structural remodeling and mitochondrial dysfunction (9, 10, 19–24). Meanwhile, muscle loss may accelerate disease progression by reducing respiratory muscle strength, impairing the body’s immune function and decreasing exercise endurance reserve. Consequently, a bidirectional relationship likely exists where muscle loss both results from and amplifies disease severity, contributing significantly to exercise limitation and functional decline (25, 26). Notably, FFMI and ASMI emerged as strong independent indicators of the risk and severity of COPD, underscoring their clinical value for risk stratification (27). Interestingly, the relative preservation of TLSM suggests a potential component specific adaptation, possibly reflecting compensatory hypertrophy of respiratory muscles, a hypothesis requiring validation through advanced imaging (28–30). This component specific adaptation may also contribute to the elevated energy expenditure observed in these patients, linking structural change to metabolic demand.

Beyond muscle depletion, we identified a dynamic and pathogenic shift in fat distribution. Systemic fat indicators (FM, FMI, BFP) in COPD patients were significantly higher than those in healthy controls and acted as risk factors for the disease, indicating a close association between systemic fat accumulation and the risk of COPD development. In contrast, local fat indicators (AFM, TFM) showed no intergroup difference, yet exhibited a decreasing trend with the elevation of GOLD stage and served as protective factors for disease severity. This seemingly contradictory phenomenon is associated with the hypermetabolic state, systemic chronic inflammation and insufficient nutritional intake in patients, and may also involve the functional redistribution of fat (31–34). During disease progression, insufficient energy intake caused by systemic hypercatabolism and decreased appetite leads to preferential depletion of appendicular and truncal fat to meet energy demands. Preserved local fat may exert a compensatory protective effect by attenuating excessive skeletal muscle breakdown through energy storage. Concurrently, systemic fat accumulation may be accompanied by functional differentiation of adipose tissue, and an increased proportion of visceral fat may represent a key pathological component. Visceral fat may accelerate disease progression via dual mechanisms: mechanically restricting diaphragmatic movement and secreting pro-inflammatory cytokines such as IL-6 and TNF-α, thereby exacerbating systemic inflammation and metabolic dysfunction (35–38). Thus, COPD-related abnormal fat metabolism is essentially pathological remodeling of fat distribution and function. Such dynamic changes in fat distribution are not only risk factors for COPD occurrence, but also act as active drivers of disease progression by inducing systemic inflammation, mediating muscle catabolism and metabolic dysfunction.

Previous studies found that the interplay between muscle depletion and pathological fat redistribution converges in a systemic hypermetabolic state, characterized by dysregulated BMR and disturbances in fluid balance (28, 31, 39). This state is sustained by a vicious cycle: The energy cost of respiratory work and systemic inflammation (28, 40), particularly pro-inflammatory cytokines secreted by visceral adipose tissue, further amplifies the inflammatory cascade (35–37, 41)—drives hypermetabolism, which in turn may exacerbate muscle wasting and energy imbalance. The pattern and magnitude of this hypermetabolic state can be further modulated by individual and environmental factors, and may exhibit sex-specific differences in inflammatory phenotype and fat distribution. In our study, we did not find statistical differences between TBW and ECW Ratio in COPD patients. However, other studies have found that BCM was significantly lower in the COPD group and changed in parallel with BMR, supporting a close association between metabolic dysfunction and impaired body cell function (42, 43), which may result from the selective depletion of peripheral skeletal muscle combined with inflammation-mediated cellular edema or water-sodium retention (44). Crucially, the intensity of this hypermetabolism appears to be severity-dependent and modulated by lifestyle factors, integrating intrinsic disease mechanisms with extrinsic influences. Thus, metabolic alteration may integrate muscle and fat abnormalities into a self-perpetuating pathological triad, but prospective studies are needed to verify this mechanism.

The main strengths and contributions of this study are as follows. First, it evaluates body composition in a multi-center cohort using sub-components of muscle mass, fat distribution, and metabolic status rather than relying on BMI alone. Second, it integrates inflammatory and functional variables, including CRP, 6MWD, HGS, and IPAQ, thereby improving the clinical interpretability of the body composition models. Third, the predictive models were adjusted for age, sex, smoking, CRP, and 6MWD, and exploratory sex-stratified analyses were provided to address sex-specific sarcopenia thresholds. Fourth, all of the above strengths support the potential use of integrated body composition assessment for clinical risk stratification, and facilitate the development of combined models to optimize severity stratification of COPD. Several limitations should be acknowledged: the cross-sectional design precludes causal inference; HGS, 6MWD, and IPAQ were incorporated from available clinical assessments and should be prospectively standardized in future studies; detailed dietary intake and nutritional intervention data were unavailable, limiting adjustment for nutritional confounding; and the models require external validation before routine clinical application.

Taken together, these findings support a shift from isolated anthropometric metrics toward multidimensional assessment of muscle, fat, metabolic, inflammatory, and functional status in COPD. This integrated approach may reduce the diagnostic ambiguity of BMI and help identify high-risk phenotypes, including sarcopenic obesity. Future longitudinal studies are warranted to elucidate the temporal dynamics of these body composition changes, and interventional trials should test the efficacy of strategies aimed at concurrently improving muscle mass, optimizing fat distribution, and modulating metabolic state to mitigate COPD progression.

## 5 Conclusion

This study suggests that an integrative muscle-fat-metabolism framework, supplemented by inflammatory and functional indicators, may improve stratification of COPD risk and severity compared with conventional metrics. These findings support the potential role of multidimensional body composition assessment in personalized COPD management, but prospective validation is required before routine implementation.

## Data Availability

Data supporting the findings of this study are available from the corresponding author upon reasonable request. The data are not publicly available due to privacy or ethical restrictions.
