# Supplementary material for: A multidimensional muscle-fat-metabolism model for severity stratification in COPD: a multi-center cross-sectional study
Source: Front Nutr. 2026 Aug 24;13:1835742. doi: 10.3389/fnut.2026.1835742 (PMC13546983; doi:10.3389/fnut.2026.1835742)
Supplement: Supplementary file 1 [file Image_1.pdf]

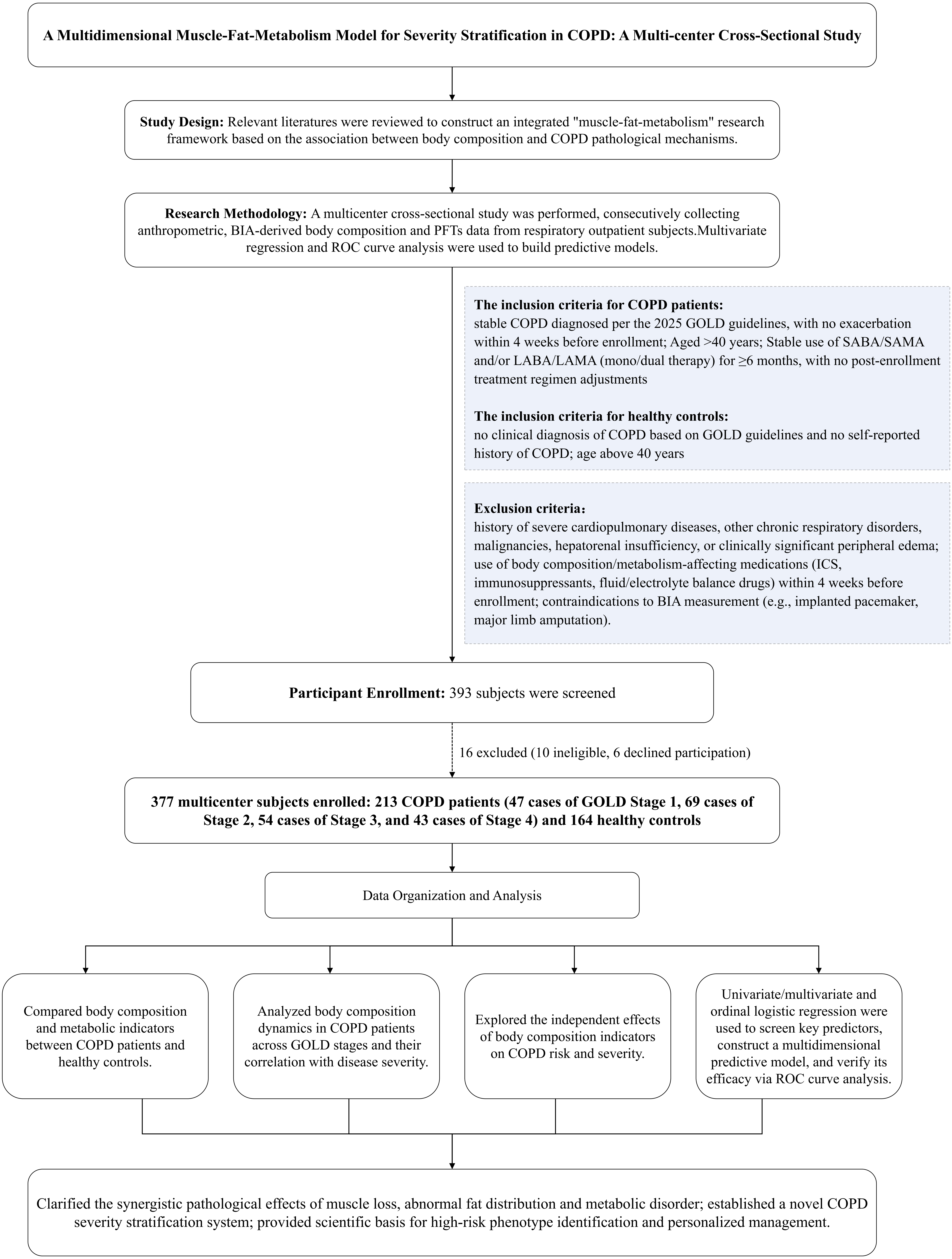
A Multidimensional Muscle-Fat-Metabolism Model for Severity Stratification in COPD: A Multi-center Cross-Sectional Study
Study Design: Relevant literatures were reviewed to construct an integrated "muscle-fat-metabolism" research framework based on the association between body composition and COPD pathological mechanisms.
Research Methodology: A multicenter cross-sectional study was performed, consecutively collecting anthropometric, BIA-derived body composition and PFTs data from respiratory outpatient subjects.Multivariate regression and ROC curve analysis were used to build predictive models.
The inclusion criteria for COPD patients:
stable COPD diagnosed per the 2025 GOLD guidelines, with no exacerbation within 4 weeks before enrollment; Aged >40 years; Stable use of SABA/SAMA and/or LABA/LAMA (mono/dual therapy) for ≥6 months, with no post-enrollment treatment regimen adjustments
The inclusion criteria for healthy controls:
no clinical diagnosis of COPD based on GOLD guidelines and no self-reported history of COPD; age above 40 years
Exclusion criteria：
history of severe cardiopulmonary diseases, other chronic respiratory disorders, malignancies, hepatorenal insufficiency, or clinically significant peripheral edema; use of body composition/metabolism-affecting medications (ICS, immunosuppressants, fluid/electrolyte balance drugs) within 4 weeks before enrollment; contraindications to BIA measurement (e.g., implanted pacemaker, major limb amputation).
Participant Enrollment: 393 subjects were screened
16 excluded (10 ineligible, 6 declined participation)
377 multicenter subjects enrolled: 213 COPD patients (47 cases of GOLD Stage 1, 69 cases of Stage 2, 54 cases of Stage 3, and 43 cases of Stage 4) and 164 healthy controls
Data Organization and Analysis
Compared body composition and metabolic indicators between COPD patients and healthy controls.
Analyzed body composition dynamics in COPD patients across GOLD stages and their correlation with disease severity.
Explored the independent effects of body composition indicators on COPD risk and severity.
Univariate/multivariate and ordinal logistic regression were used to screen key predictors, construct a multidimensional predictive model, and verify its efficacy via ROC curve analysis.
Clarified the synergistic pathological effects of muscle loss, abnormal fat distribution and metabolic disorder; established a novel COPD severity stratification system; provided scientific basis for high-risk phenotype identification and personalized management.
