# Supplementary material for: A multidimensional muscle-fat-metabolism model for severity stratification in COPD: a multi-center cross-sectional study
Source: Front Nutr. 2026 Aug 24;13:1835742. doi: 10.3389/fnut.2026.1835742 (PMC13546983; doi:10.3389/fnut.2026.1835742)
Supplement: Supplementary file 2 [file Table_1.docx]

**A Multidimensional Muscle-Fat-Metabolism Model for Severity Stratification in COPD: A Multi-center Cross-Sectional Study**

**Supplementary Tables**

**Supplementary Table 1** ***Evaluation of Predictive Efficacy of Body Composition Indicators for Risk of COPD Occurrence***

| No | Model | AUC（95%CI） | Sensitivity | Specificity | Cutoff values | Youden index | AIC |
| --- | --- | --- | --- | --- | --- | --- | --- |
| 1 | Clinical Baseline | 0.747 (0.698-0.796) | 0.653 | 0.720 | 0.579 | 0.372 | 454.018 |
| 2 | Composition Distribution | 0.839 (0.800-0.879) | 0.737 | 0.817 | 0.592 | 0.554 | 392.098 |
| 3 | Composition Metabolism | 0.867 (0.831-0.903) | 0.779 | 0.823 | 0.594 | 0.603 | 362.885 |
| 4 | Classification | 0.825 (0.784-0.867) | 0.728 | 0.805 | 0.584 | 0.533 | 401.645 |

***Note***:

Model 1 (Clinical Basic Model) included sex, age, smoking, CRP, 6MWD, WC and BMI;
Model 2 (Composition Distribution Model) included sex, age, smoking, CRP, 6MWD, FFMI and WC;
Model 3 (Composition Metabolism Model) included sex, age, smoking, CRP, 6MWD, FFMI, WC and BMR;
Model 4 (Classification Model) included sex, age, smoking, CRP, 6MWD, Low ASMI, High VFA and BMR.

***Abbreviations***: AUC: Area under the curve; CI: Confidence interval; AIC: Akaike information criterion index; CRP: C-Reactive Protein; 6MWD: 6-Minute Walk Distance; BMI: Body mass index; WC: Waist circumference; FFMI: Fat free mass index; BMR: Basal metabolic rate; ASMI: Appendicular skeletal muscle mass index; VFA: Visceral fat area.

**Supplementary Table 2 *Evaluation of Predictive Efficacy of Body Composition Indicators for COPD-GOLD Grading by Macro-Average ROC Analysis***

| Model | GOLD | AUC | 95%CI | Macro-Average AUC | sensitivity | specificity | cutoff values | Youden’s index |
| --- | --- | --- | --- | --- | --- | --- | --- | --- |
| Model 1  COPD Prediction Models | 1 | 0.848 | 0.780 - 0.916 | 0.812 | 0.889 | 0.380 | 0.148 | 0.269 |
|  | 2 | 0.793 | 0.713 - 0.873 |  |  |  |  |  |
|  | 3 | 0.711 | 0.630 - 0.793 |  |  |  |  |  |
|  | 4 | 0.897 | 0.823 - 0.951 |  |  |  |  |  |
| Model 2  Muscle-Fat Model | 1 | 0.866 | 0.801 - 0.931 | 0.808 | 0.373 | 0.900 | 0.426 | 0.273 |
|  | 2 | 0.793 | 0.718 - 0.869 |  |  |  |  |  |
|  | 3 | 0.641 | 0.555 - 0.726 |  |  |  |  |  |
|  | 4 | 0.933 | 0.888 - 0.978 |  |  |  |  |  |
| Model 3  Muscle-Fat Metabolism Model | 1 | 0.867 | 0.801 - 0.933 | 0.811 | 0.663 | 0.590 | 0.237 | 0.253 |
|  | 2 | 0.793 | 0.717 - 0.869 |  |  |  |  |  |
|  | 3 | 0.638 | 0.552 - 0.724 |  |  |  |  |  |
|  | 4 | 0.947 | 0.905 - 0.989 |  |  |  |  |  |

**Note:** Youden's index: the cutoff value corresponding to the maximum sum of sensitivity and specificity.
Model 1 (COPD Occurrence Prediction Model): sex, age, smoking, CRP, 6MWD, FFMI, WC and BMR;
Model 2 (Muscle-Fat Model): sex, age, smoking, CRP, 6MWD, ASMI and BFP;
Model 3 (Muscle-Fat Metabolism Model): sex, age, smoking, CRP, 6MWD, ASMI, BFP and BMR.

***Abbreviations***: GOLD: Global initiative for chronic obstructive lung disease; AUC: Area under the curve; CI: Confidence interval; CRP: C-Reactive Protein; 6MWD: 6-Minute Walk Distance; WC: Waist circumference; FFMI: Fat free mass index; BMR: Basal metabolic rate; ASMI: Appendicular skeletal muscle mass index; BFP: Body fat percentage

**Supplementary Table 3 *Multivariate Logistic Regression Analysis of Body Composition Indicators for COPD Occurrence, stratified by Sex.***

| Variables | *Multivariate logistic regression analysis (adjusted for age, smoking, CRP and* 6MWD*)* | | | | | |
| --- | --- | --- | --- | --- | --- | --- |
|  | β | S.E | Z | *P* | OR (95%CI) | VIF |
| ***Male*** | | | | | | |
| *Model 1: Clinical Baseline Model* | | | | | | |
| BMI | -0.290 | 0.074 | -3.917 | <0.001 | 0.748 (0.647 ~ 0.865) | 3.895 |
| WC | 0.094 | 0.032 | 2.930 | 0.003 | 1.098 (1.032 ~ 1.170) | 3.941 |
| *Model 2: Composition Distribution Model* | | | | | | |
| FFMI | -0.662 | 0.105 | -6.321 | <0.001 | 0.516 (0.420 ~ 0.633) | 1.342 |
| WC | 0.076 | 0.023 | 3.317 | <0.001 | 1.079 (1.032 ~ 1.129) | 1.390 |
| *Model 3: Composition Metabolism Model* | | | | | | |
| FFMI | -1.116 | 0.158 | -7.086 | <.001 | 0.327 (0.240 ~ 0.446) | 2.932 |
| WC | 0.058 | 0.023 | 2.487 | 0.013 | 1.060 (1.012 ~ 1.110) | 1.446 |
| BMR | 0.009 | 0.002 | 4.375 | <.001 | 1.009 (1.005 ~ 1.013) | 3.289 |
| *Model 4: Classification Model* | | | | | | |
| Low ASMI | 2.783 | 0.417 | 6.667 | <.001 | 16.173 (7.136 ~ 36.657) | 1.696 |
| High VFA | 0.51 | 0.34 | 1.501 | 0.133 | 1.666 (0.856 ~ 3.244) | 1.060 |
| BMR | 0.003 | 0.001 | 2.239 | 0.025 | 1.003 (1.001 ~ 1.006) | 1.875 |
| ***Female*** | | | | | | |
| *Model 1: Clinical Baseline Model* | | | | | | |
| BMI | 0.25 | 0.2 | 1.22 | 0.221 | 1.28 (0.86 ~ 1.91) | 12.947 |
| WC | -0.09 | 0.11 | -0.87 | 0.387 | 0.91 (0.73 ~ 1.13) | 12.387 |
| *Model 2: Composition Distribution Model* | | | | | | |
| FFMI | -0.81 | 0.32 | -2.55 | 0.011 | 0.45 (0.24 ~ 0.83) | 3.093 |
| WC | 0.17 | 0.06 | 2.63 | 0.008 | 1.18 (1.04 ~ 1.34) | 2.712 |
| *Model 3: Composition Metabolism Model* | | | | | | |
| FFMI | -1.54 | 0.44 | -3.53 | <.001 | 0.21 (0.09 ~ 0.50) | 4.664 |
| WC | 0.13 | 0.06 | 2.18 | 0.030 | 1.14 (1.01 ~ 1.29) | 2.906 |
| BMR | 0.02 | 0.01 | 3.02 | 0.003 | 1.02 (1.01 ~ 1.03) | 3.541 |
| *Model 4: Classification Model* | | | | | | |
| Low ASMI | 2.06 | 0.8 | 2.57 | 0.010 | 7.85 (1.63 ~ 37.77) | 1.760 |
| High VFA | 0.43 | 0.61 | 0.71 | 0.475 | 1.54 (0.47 ~ 5.05) | 1.285 |
| BMR | 0.01 | 0 | 2.31 | 0.021 | 1.01 (1.01 ~ 1.02) | 2.032 |

***Abbreviations***: CRP: C-Reactive Protein; 6MWD: 6-Minute Walk Distance; OR: Odds ratio; CI: Confidence interval; VIF: Variance inflation factor; BMI: Body mass index; WC: Waist circumference; FFMI: Fat free mass index; BMR: Basal metabolic rate; ASMI: Appendicular skeletal muscle mass index; VFA:Visceral fat area.

**Supplementary Table 4. *Multivariate Ordinal Logistic Regression Analysis of Body Composition Indicators for COPD Severity (GOLD 1~4) , stratified by Sex.***

| Variables | *Multivariate ordinal logistic regression analysis (**adjusted for age, smoking, CRP and* 6MWD *)* | | | | | |
| --- | --- | --- | --- | --- | --- | --- |
|  | β | S.E | t | *P* | OR (95%CI) | VIF |
| ***Male*** | | | | | | |
| *Composition Metabolism Model* | | | | | | |
| FFMI | -0.5 | 0.19 | -2.69 | **0.007** | 0.60 (0.42 ~ 0.87) | 3.182 |
| WC | 0.03 | 0.02 | 1.12 | 0.261 | 1.03 (0.98 ~ 1.08) | 1.700 |
| BMR | 0 | 0 | 1.12 | 0.263 | 1.00 (1.00 ~ 1.01) | 3.363 |
| *Muscle-Fat Model* | | | | | | |
| ASMI | -0.74 | 0.15 | -5.02 | **<.001** | 0.48 (0.36 ~ 0.64) | 1.090 |
| BFP | 0.05 | 0.03 | 1.93 | 0.053 | 1.05 (1.00 ~ 1.11) | 1.094 |
| *Muscle-Fat-Metabolism Model* | | | | | | |
| ASMI | -1.25 | 0.46 | -2.7 | **0.007** | 0.29 (0.12 ~ 0.71) | 2.029 |
| BFP | 0.05 | 0.03 | 1.75 | 0.08 | 1.05 (0.99 ~ 1.11) | 1.116 |
| BMR | 0 | 0 | 1.62 | 0.106 | 1.00 (1.00 ~ 1.01) | 2.175 |
| ***Female*** | | | | | | |
| *Composition Metabolism Model* | | | | | | |
| FFMI | -0.71 | 0.55 | -1.31 | 0.19 | 0.49 (0.17 ~ 1.42) | 5.841 |
| WC | 0.06 | 0.06 | 0.86 | 0.389 | 1.06 (0.93 ~ 1.20) | 2.936 |
| BMR | 0.01 | 0.01 | 0.88 | 0.378 | 1.01 (0.99 ~ 1.02) | 4.030 |
| *Muscle-Fat Model* | | | | | | |
| ASMI | -0.68 | 0.4 | -1.71 | 0.088 | 0.50 (0.23 ~ 1.11) | 1.364 |
| BFP | 0.05 | 0.07 | 0.69 | 0.493 | 1.05 (0.91 ~ 1.21) | 1.439 |
| *Muscle-Fat-Metabolism Model* | | | | | | |
| ASMI | -3.5 | 1.45 | -2.42 | **0.016** | 0.03 (0.00 ~ 0.51) | 1.801 |
| BFP | 0.09 | 0.08 | 1.21 | 0.226 | 1.10 (0.94 ~ 1.27) | 1.441 |
| BMR | 0.02 | 0.01 | 2.85 | **0.004** | 1.02 (1.01 ~ 1.04) | 1.443 |

***Abbreviations***: CRP: C-Reactive Protein; 6MWD: 6-Minute Walk Distance; OR: Odds ratio; CI: Confidence interval; VIF: Variance inflation factor; FFMI: Fat free mass index; WC: Waist circumference; BMR: Basal metabolic rate; ASMI: Appendicular skeletal muscle mass index; BFP: Body fat percentage.
